# Temperature dependence of the superconducting energy gaps in Ca_9.35_La_0.65_(Pt_3_As_8_)(Fe_2_As_2_)_5_ single crystal

**DOI:** 10.1038/s41598-018-24940-9

**Published:** 2018-06-05

**Authors:** Yu-il Seo, Woo-jae Choi, D. Ahmad, Shin-ichi Kimura, Yong Seung Kwon

**Affiliations:** 1 Department of Emerging Materials Science, DGIST, Daegu, 711-873 Republic of Korea; 2 Graduate School of Frontier Biosciences and Department of Physics, Graduate School of Science, Osaka University, Suita, 565-0871 Japan

## Abstract

We measured the optical reflectivity *R*(*ω*) for an underdoped (Ca_0.935_La_0.065_)_10_(Pt_3_As_8_)(Fe_2_As_2_)_5_ single crystal and obtained the optical conductivity ${\sigma }_{1}(\omega )$ using the K-K transformation. The normal state ${\sigma }_{1}(\omega )$ at 30 K is well fitted by a Drude-Lorentz model with two Drude components (*ω*_*p*1_ = 1446 cm^−1^ and *ω*_*p*2_ = 6322 cm^−1^) and seven Lorentz components. Relative reflectometry was used to accurately determine the temperature dependence of the superconducting gap at various temperatures below *T*_c_. The results clearly show the opening of a superconducting gap with a weaker second gap structure; the magnitudes for the gaps are estimated from the generalized Mattis-Bardeen model to be Δ_1_ = 30 and Δ_2_ = 50 cm^−1^, respectively, at *T* = 8 K, which both decrease with increasing temperature. The temperature dependence of the gaps was not consistent with one-band BCS theory but was well described by a two-band (hence, two gap) BCS model with interband interactions. The temperature dependence of the superfluid density is flat at low temperatures, indicating an *s*-wave full-gap superconducting state.

## Introduction

Since the discovery of superconductivity with *T*_c_ = 26 K in LaFeAsO_1−x_F_x_^1^, many productive efforts have been made to find other new iron-based superconductors, which have led to the discovery of several families of superconductors, including the so-called 1111, 122, 111, 11 and 42622 families^1–5^. Recently, new iron-based superconductor compounds Ca_10_(Pt_n_As_8_)(Fe_2_As2)_5_ (n = 3, 4)^6–9^ with planar Pt_n_As_8_ (n = 3, 4) were discovered and added as yet another family of iron-based superconductors. Both compounds share a similar overall crystal structure consisting of a tetrahedral FeAs layer sandwiched between Pt_n_As_8_ intermediary layers but show distinct differences in their details: the Ca_10_(Pt_4_As_8_)(Fe_2_As_2_)_5_ compound crystallizes in a tetragonal crystal structure and exhibits metallic behaviour. Additionally, the compound becomes superconducting with a maximum *T*_c_ of 38 K^7^ without any doping, with electron doping causing a reduction in the superconducting transition temperature^8^. On the other hand, the parent compound Ca_10_(Pt_3_As_8_)(Fe_2_As_2_)_5_ shows a triclinic crystal structure that becomes semiconducting and antiferromagnetically ordered below *T*_N_ ~ 120 K without any reduction in the crystal symmetry^10^. Electron doping using La and Pt substitution for the Ca and Fe sites can induce superconductivity and a semiconductor-metal transition, as well as the suppression of antiferromagnetic ordering, with a maximum *T*_c_ for each substitution of ~32 K and ~15 K, respectively^11–13^.

Differences are also suggested by theoretical band calculations as well as experimentally, such as by angle-resolved photoemission spectroscopy (ARPES) measurements. According to a former suggestion, such differences arise from the difference in the metallicity of the Pt_n_As_8_ layers^14^, with the latter suggestion that the differences can develop from the number of band-edge singularities^15^. To investigate the effect of such subtle differences on the electronic structure and superconductivity, various experimental studies based on transport^11^, pressure effects^16^, NMR^17^, ARPES^18^, neutron^19^, and IR spectroscopy^12^ have been carried out; thus, much progress has been made. However, detailed studies on the characteristic changes of superconducting gaps due to such differences in the electronic structure have rarely been carried out, with the in-depth understanding of the superconductivity mechanism, as well as the systematic development of superconductivity, having hardly progressed.

In this paper, to study the fine temperature dependence of the superconducting gaps for an electron-underdoped (Ca_0.935_La_0.065_)_10_(Pt_3_As_8_)(Fe_2_As_2_)_5_ single crystal, we measured the single crystal reflectivity in the frequency range from 20 to 600 cm^−1^ at various temperatures below *T*_c_ using a reflectance measurement method that measures the relative reflectivity with respect to a temperature change in addition to the absolute reflectivity. The reflectivity spectra measured by this method in addition to the optical spectra calculated by the Kramers-Kronig (K-K) transformation show an apparent gap opening at 60 cm^−1^ and a weak second gap structure at ~100 cm^−1^ for a temperature of 8 K. As the temperature increases, the magnitude of the superconducting gap decreases slowly at low temperatures but decreases rapidly near *T*_c_. The temperature-dependent feature in both the gaps was qualitatively described by a two-band BCS model with interband interactions^20^. The superfluid density analysis showed that the underdoped (Ca_0.935_La_0.065_)_10_(Pt_3_As_8_)(Fe_2_As_2_)_5_ compound exhibits a superconducting state with an *s*-wave full gap.

## Results and Discussion

**Figure 1:**
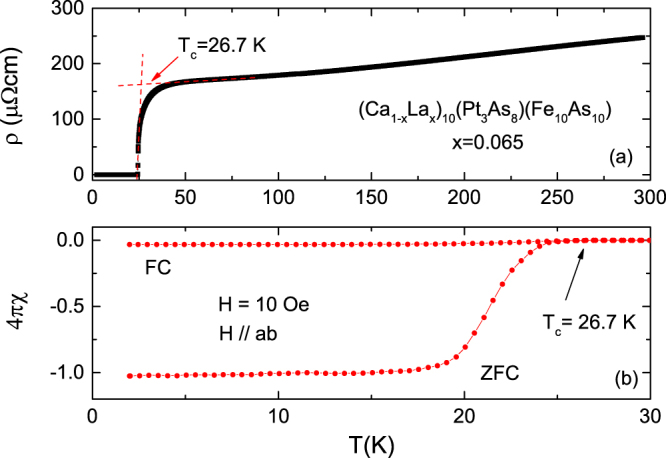
Temperature dependence of the electrical resistivity (**a**) and magnetic susceptibility (**b**) for the (Ca_0.935_La_0.065_)_10_(Pt_3_As_8_)(Fe_2_As_2_) single crystal.

Figure 1(a,b) show the temperature dependence of the electrical resistivity and magnetization for a (Ca_0.935_La_0.065_)_10_(Pt_3_As_8_)(Fe_2_As_2_)_5_ single crystal, respectively. The magnetization was measured in the *H*//*ab*-plane with *H* = 20 Oe. The electrical resistivity decreases almost linearly with temperature down to 100 K and then tends to saturate but becomes zero at ~25 K. As shown in the figure, *T*_c_ is 26.7 K, as determined by the criterion being the temperature at which the extension line of the normal state meets the extension line of the electrical resistivity in the superconducting state. As shown in Fig. 1(b), the ZFC (zero-field cooling) magnetic susceptibility $4{\rm{\pi }}\chi \,\,$is reduced to ~−1, indicating the bulk nature of the superconductivity resulting from the complete screening of the interior grains, with the small values of $4{\rm{\pi }}\chi$ in FC (field cooling) indicating weak flux pinning of the crystal under a low applied field.

**Figure 2:**
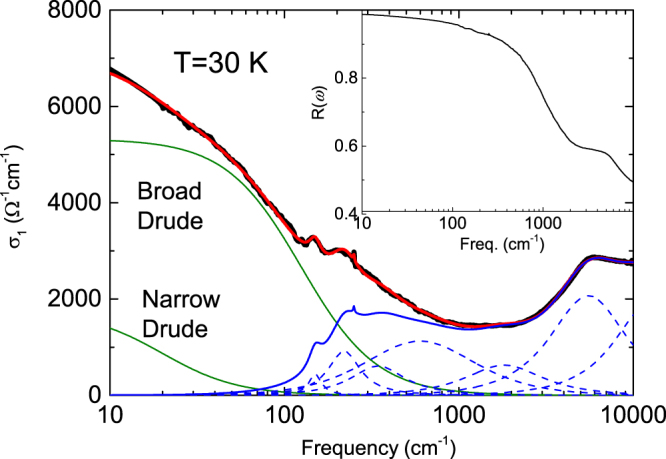
Optical conductivity ${\sigma }_{1}(\omega )$ for the (Ca_0.935_La_0.065_)_10_(Pt_3_As_8_)(Fe_2_As_2_) single crystal at 30 K (normal state) on a semi-logarithm scale. In this figure, the green lines show fits to a Drude response, and the navy dashed and solid lines show fits to Lorentz oscillators and the sum of the Lorentz oscillators, respectively. The inset shows the measured reflectivity data for the (Ca_0.935_La_0.065_)_10_(Pt_3_As_8_)(Fe_2_As_2_) single crystal at 30 K.

The inset of Fig. 2 shows the reflectivity spectrum *R*(*ω*) for the (Ca_0.935_La_0.065_)_10_(Pt_3_As_8_)(Fe_2_As_2_)_5_ single crystal at *T* = 30 K (normal state) on a semi-logarithm scale. The reflectivity tends to approach unity at low frequencies, indicating metallic behaviour for the crystal, and shows peak structures due to interband transitions at high frequencies. To carry out a more conventional analysis of the optical properties, we derived *σ*(*ω*) from the *R*(*ω*) spectra through a K-K transformation. In the K-K transformation, the reflectivity was extrapolated using a Hagen-Rubens function below 20 cm^−1^, a constant reflectivity from ~1.5 eV (=12000 cm^−1^) to 40 eV and then a free-electron approximation *R*(*ω*) ∝ *ω*^−4^. The optical conductivity ${\sigma }_{1}(\omega )$ obtained from the reflectivity data presented in the inset is shown in the main panel of Fig. 2. The optical conductivity shows a broad Drude peak centred at *ω* = 0, indicating a metallic character, and then decreases with increasing frequency until the hump due to interband transitions beings to appear at approximately 2000 cm^−1^. However, the decrease is not monotonic, but instead, two far-infrared peaks in the frequency region from 150 to 230 cm^−1^ occur on the decreasing side. Furthermore, a sharp peak due to an optically active phonon is observed at 250 cm^−1^.

According to first-principle calculations for Ca_10_(Pt_3_As_8_)(Fe_2_As_2_)_5_, the density of states (DOS) in the vicinity of *E*_F_ is mainly dominated by the Fe 3*d* orbitals^18^. The 3d orbitals were also observed in the ARPES experiment; hole-like *α*_1_ and *α*_2_ pockets around the zone centre as well as two electron-like ${\rm{\beta }}$ pockets at the zone corner in the parent compound Ca_10_(Pt_3_As_8_)(Fe_2_As_2_)_5_ were clearly observed, while for the optimally Pt-doped sample, the *α*_1_ and the two β (though not clearly resolved) Fermi pockets remained, but the ${\alpha }_{2}$ band moved just below *E*_F_^15^. Moreover, the top of a hole-like ${\alpha }_{3}$ band dispersion was also observed at approximately 600 cm^−1^ below *E*_F_. Compared to the ARPES results, our two peak structures observed at approximately 200 cm^−1^ appear to be due to absorptions from these bands. On the other hand, broad Pt-5d bands are observed at 0.2–0.3 eV below *E*_F_ in the band calculation and the ARPES results; however, in our results, no absorption due to the bands in the form of a prominent peak was observed. Similar low-frequency interband transitions were discussed for Ba(Fe_1−x_Co_x_)_2_As_2_^21^.

For quantitative analysis of the optical conductivity due to conduction and bound electrons, we attempted to fit the optical conductivity using the standard Drude-Lorentz model:

1 $${\sigma }_{1}(\omega )=\,\frac{1}{4\pi }\,Re[\sum _{j}\frac{{\omega }_{p,Dj}^{2}}{\frac{1}{{\tau }_{Dj}}-i\omega }+\,\sum _{k}{S}_{k}\frac{\omega }{\frac{\omega }{{\tau }_{Lk}}+i({\omega }_{Lk}^{2}-{\omega }^{2})}],$$

where ${\omega }_{p,Dj}$ and $1/{\tau }_{Dj}$ are the plasma frequency and scattering rate for the *j*th free carrier Drude band, respectively, and ${S}_{k}$, ${\omega }_{Lk}$ and $1/{\tau }_{Lk}$ are the spectral weight, resonance frequency, and scattering rate of the *k*th oscillator, respectively.

The fitting result for the optical conductivity data measured at 30 K using the Drude-Lorentz model is plotted in the main panel of Fig. 2. The optical conductivity is well fitted by a Drude-Lorentz model with two Drude components and seven Lorentz oscillator components. The fact that our sample is well described by two Drude components indicates the existence of multiple bands, as is the case for many IBS compounds^22–28^. The plasma frequencies (corresponding scattering rates) were evaluated to be ${\omega }_{p1}$ = 1446 cm^−1^ ($1/{\tau }_{D1}$ = 20 cm^−1^) for the narrow Drude band and ${\omega }_{p2}$ = 6322 cm^−1^ ($1/{\tau }_{D2}$ = 125 cm^−1^) for the broad Drude band, respectively. The high-frequency interband transitions are explained by two Lorentz oscillator components, similar to the results for Ca_8.5_La_1.5_(Pt_3_As_8_)(Fe_2_As_2_)_5_ reported previously^12^, with the far-infrared interband transitions well fitted by two Lorentz oscillator components at 150 and 230 cm^−1^. In addition, the transition from the Pt 5*d* band mentioned above was included in this fitting using a broad Lorentz oscillator at approximately 2000 cm^−1^, with the transitions due to the broad ${\alpha }_{3}$ band included using two relatively broad Lorentz oscillators at approximately 300 and 600 cm^−1^.

**Figure 3:**
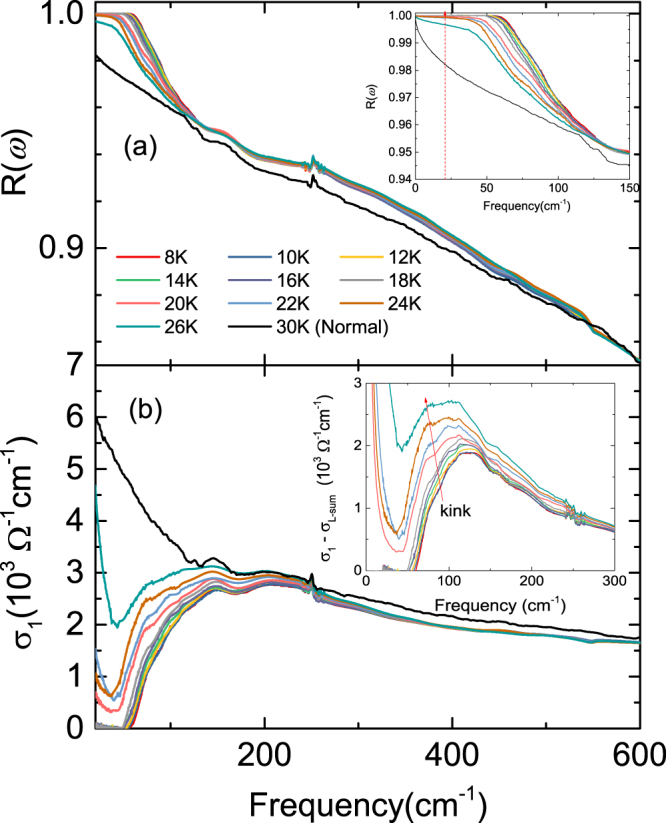
Measured reflectivity spectra *R*(*ω*) (**a**) and determined ${\sigma }_{1}(\omega )$ (**b**) for the (Ca_0.935_La_0.065_)_10_(Pt_3_As_8_)(Fe_2_As_2_) single crystal at varying temperatures ranging from 8 to 26 K below *T*_c_ and 30 K (normal state) in the low frequency region below 600 cm^−1^. The inset in the upper panel shows an enlarged view of the reflectivity spectra below 150 cm^−1^, and the inset in the lower panel shows the optical conductivity following removal of the sum of the Lorentz oscillators.

Figure 3 (a) shows the reflectivity spectra *R*(*ω*) for the (Ca_0.935_La_0.065_)_10_(Pt_3_As_8_)(Fe_2_As_2_)_5_ single crystal measured at varying temperatures ranging from 8 K to 30 K. The reflectivity spectra up to 26 K (superconducting state) were measured by the relative reflectivity measurement (RRM) method. The data at 30 K in this figure correspond to those shown in Fig. 2, which were measured by the absolute reflectivity measurement (ARM) method. The main panel (a) shows the full data measured using the RRM method for frequencies ranging from 20 to 600 cm^−1^, with the inset showing a close-up view of the data from 0 to 150 cm^−1^; here, the data from 0 to 20 cm^−1^ (frequency region below the red dotted line) are not measured but extrapolated, as explained below. The low-frequency reflectivity data in the superconducting state show a sharp rise below 120 cm^−1^ before reaching a flat unity response below ~60 cm^−1^, indicating that the superconducting gap is open. As the temperature increases towards *T*_c_, the flat unity response shifts to lower frequencies as the superconducting gap size is reduced. The shape of the low-frequency reflectivity and its temperature dependence are a clear signature of the fully open superconducting gap.

Figure 3(b) shows the optical conductivity spectra ${\sigma }_{1}(\omega )$ obtained from the data presented in Fig. 3(a) in the frequency range from 0 to 600 cm^−1^ for varying temperatures below 30 K. Compared to the optical conductivity of the normal state, ${\sigma }_{1}(\omega )$ in the superconducting state is strongly suppressed in the low-frequency region and shows peaks at ~150 cm^−1^. ${\sigma }_{1}(\omega )$ for *T* < 20 K is perfectly suppressed at low frequencies below 60 cm^−1^; this frequency slowly decreases as the temperature increases, showing a clear absorption edge, which reflects the superconducting gap opening. In the superconducting state at *T* > 20 K, ${\sigma }_{1}(\omega )$ is suppressed but does not become zero near the absorption edge and increases again in the lower frequency region. The shape of the absorption edge and its temperature dependence are another signature of the fully open superconducting gap.

**Figure 4:**
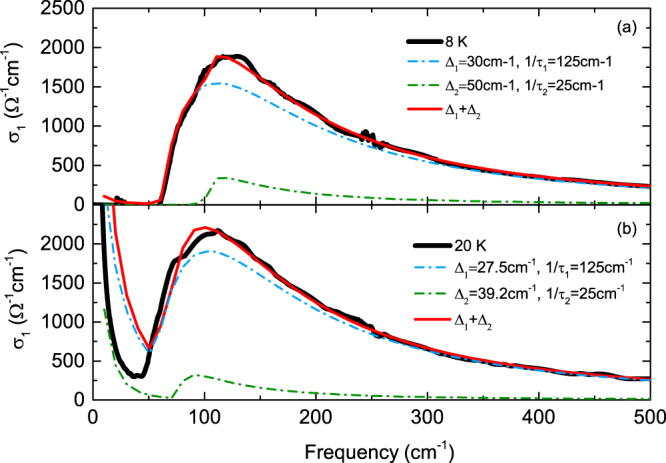
The optical conductivity at *T* = 8 (**a**) and 20 K (**b**) fitted to a generalized Mattis-Bardeen model^29^. The black solid line shows the superconducting optical conductivity after subtracting the Lorentz oscillator contribution at 30 K from the optical conductivity measured at 8 and 20 K. The blue and green dash-dotted lines show the optical conductivity determined for the smaller superconducting gap and larger gap using the Mattis-Bardeen model, respectively. The red solid line denotes the sum of the two fitted optical conductivities for the two gaps.

**Figure 5:**
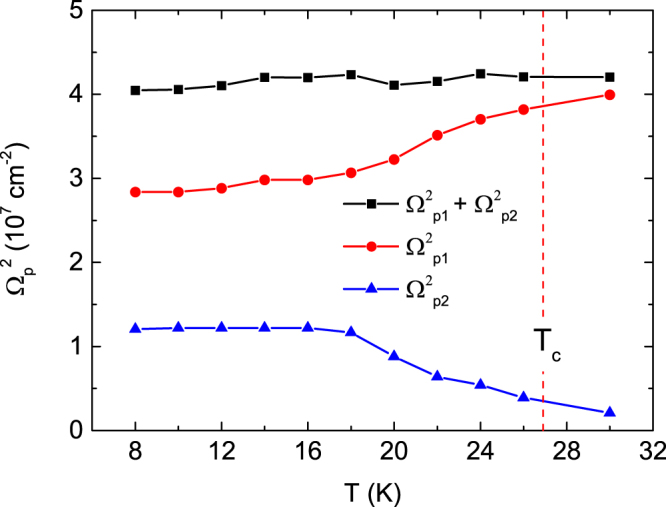
The Drude weight (${({{\rm{\Omega }}}_{p1}^{N})}^{2}\,$and ${({{\rm{\Omega }}}_{p2}^{N})}^{2}$) for the broad and narrow bands in the superconducting state, respectively, which is the fitting parameter used in a generalized Mattis-Bardeen model^29^.

Figure 4(a,b) shows the superconducting optical conductivity (black solid line) after subtraction of the Lorentz oscillator contribution determined at 30 K from the optical conductivity measured at 8 and 20 K (shown in Fig. 3(b)), respectively. The data at 8 K show a clear absorption edge at approximately 60 cm^−1^, which corresponds to the first superconducting gap size (∼$2{{\rm{\Delta }}}_{1}$), and a weak kink at approximately 100 cm^−1^ (see Fig. 3(b)), indicating the existence of a second superconducting gap. Below 60 cm^−1^, the optical conductivity remains at zero. In the high temperature regime, at 20 K, the absorption edge shifts to a lower frequency, 50 cm^−1^, with the kink also shifting to 70 cm^−1^. Below 50 cm^−1^, the optical conductivity increases again. To estimate the magnitude of the superconducting gaps, the optical conductivity data for each temperature were fitted to a generalized Mattis-Bardeen model^29^. For instance, as shown in Fig. 4, the data at 8 K are well fitted with ${{\rm{\Delta }}}_{1}=30\,{{\rm{cm}}}^{-1}$ ($1/{\tau }_{1}=125\,\,c{m}^{-1})$ and ${{\rm{\Delta }}}_{2}=50\,{{\rm{cm}}}^{-1}$ ($1/{\tau }_{2}=25\,c{m}^{-1})$ over a wide frequency region, while the data for 20 K are well fitted with ${{\rm{\Delta }}}_{1}=27.5\,{{\rm{cm}}}^{-1}$ $(1/{\tau }_{1}=125\,c{m}^{-1})$ and ${{\rm{\Delta }}}_{2}=39.2\,{{\rm{cm}}}^{-1}$ ($1/{\tau }_{2}=25\,\,c{m}^{-1})$ for frequencies above 50 cm^−1^; however, a deviation between the two sets of data occurs below 50 cm^−1^. This inconsistency comes from the uncertainty in the low-frequency extrapolations; thus, to solve this problem of inconsistency, lower-frequency data are required. The gap magnitudes at 8 K are smaller than those for the optimally La-doped 10-3-8 compound^12^; the magnitude of the smaller gap is reduced by 25%, while that of the larger gap is reduced by 60%. The scattering rates corresponding to the smaller (larger) gaps are similar to those for the broad (narrow) Drude bands shown in Fig. 2, which indicates that the smaller (larger) superconducting energy gap of ${{\rm{\Delta }}}_{1}({{\rm{\Delta }}}_{2})$ is opened at the Fermi surface of the broad (narrow) Drude bands with a larger (smaller) plasma frequency, which is consistent with ${s}^{\pm }\,$- wave theory^30^. Furthermore, the smaller gap is in the dirty limit because $\frac{1}{{\tau }_{1}} > 2{{\rm{\Delta }}}_{1}$, which results in a distinct absorption edge at approximately 60 cm^−1^, while the larger gap is close to the clean limit because $\frac{1}{{\tau }_{2}} < 2{{\rm{\Delta }}}_{2}$, which results in a blurred absorption edge at approximately 100 cm^−1^ ^31^. These fittings depend on the Drude plasma-frequency (${{\rm{\Omega }}}_{p}^{N})$ as well as on the scattering rate and superconducting gap size described above. The two scattering rates of 1/τ_1_ and 1/τ_2_ were constant, as seen above, for the temperature change. However, the two Drude weights showed a temperature dependence, as shown in Fig. 5. The ${({{\rm{\Omega }}}_{p1}^{N})}^{2}$ corresponding to the small superconducting gap shows an almost constant value up to approximately 18 K; however, it increases at higher temperatures and approaches a normal state value. On the other hand, the ${({{\rm{\Omega }}}_{p2}^{N})}^{2}$ corresponding to the large superconducting gap is constant up to 18 K (${({{\rm{\Omega }}}_{p2}^{N})}^{2}\,$is smaller than ${({{\rm{\Omega }}}_{p1}^{N})}^{2}$); however, it decreases above 18 K and approaches a normal state value. The temperature dependence of the two Drude weights indicates that the carrier densities for the two bands change due to interband interactions in the superconducting state. This result is consistent with the results we will discuss later. The sum of the two Drude weights, ${({{\rm{\Omega }}}_{p1}^{N})}^{2}+{({{\rm{\Omega }}}_{p2}^{N})}^{2}$_,_ is almost constant for the temperature change. This implies that the normal state Drude term is transferred to the particles participating in the Mattis-Bardeen superconducting response.

**Figure 6:**
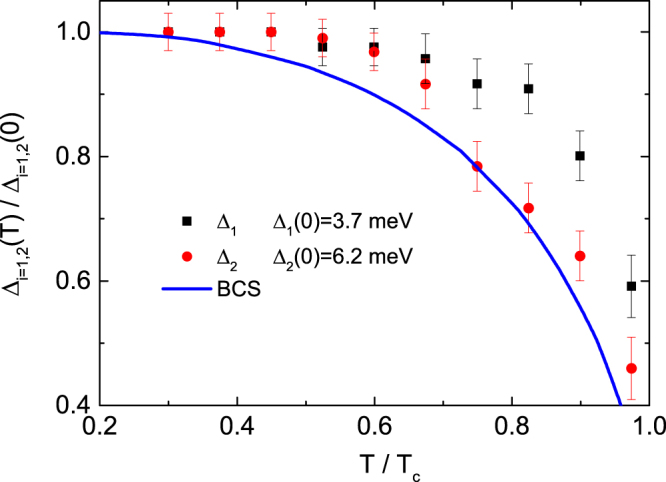
The temperature dependence of the superconducting energy gap normalized by the superconducting energy gap at *T* = 0 for the two superconducting gaps. The blue solid line shows the temperature dependence of the magnitude of the superconducting gap determined using BCS theory^32^.

As the temperature increases towards *T*_c_, the two superconducting gap sizes decrease, but the scattering rates corresponding to the gaps remain unchanged. The temperature dependence of the two superconducting gaps normalized by the superconducting gap size extrapolated to *T* = 0 K using the low-temperature superconducting gap data is plotted in Fig. 6. The magnitude of the two gaps is almost unchanged up to *T*/*T*_c_ ~ 0.5 but rapidly decreases above 0.5. The temperature dependence of the two gaps quantitatively deviates from the result obtained from one-band BCS theory, especially at higher temperatures (see Fig. 6). Interestingly, the two superconducting gaps are closed at the same transition temperature, indicating that the interband interaction is important for forming Cooper pairs^20^.

**Figure 7:**
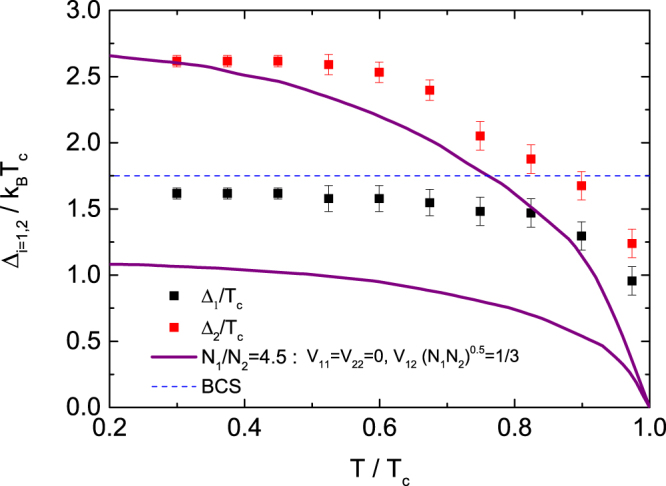
The temperature dependence of the ratios of the gap magnitude to *T*_c_, $RG={\rm{\Delta }}/{k}_{B}{T}_{c}$, for the two superconducting gaps. The purple solid lines show the ratio RG determined from a modified BCS model taking into consideration the interband interaction in a two-band system. The dashed line represents the value of *RG* = 1.75 derived from BCS theory^32^.

Figure 7 shows the temperature dependence of the ratio of the gap magnitude to *T*_c_, $RG={\rm{\Delta }}/{k}_{B}{T}_{c}$. The *RG*s extrapolated to *T* = 0 K were evaluated as approximately 1.6 and 2.6 for the smaller and larger gaps, respectively. Compared to the value of *RG* = 1.75 derived from one-band BCS theory^32^, the former is small, while the latter is very large. Such a trend is well described as a two-band BCS model with interband interactions^20^. The temperature dependence of *RG*(*T*) for the two superconducting gaps calculated by the two-band BCS model with only interband interaction is plotted as purple solid lines in Fig. 7. The *RG*(*T*) for the larger gap is roughly consistent with the line in the low temperature regime but deviates towards a larger value at high temperatures, which indicates that the experimentally determined gap opening is faster than that obtained from calculation due to strongly coupled pairs. On the other hand, the *RG*(*T*) for the smaller gap is larger than that obtained from calculation for all temperatures, indicating that intraband interaction, as well as interband interaction, plays an important role in the formation of this superconducting gap with Δ_1_.

As shown in Fig. 3, the optical conductivity was decomposed into two Drude terms, accounting for the free carrier response, and seven Lorentz terms, among which six Lorentz terms account for the interband transitions and one Lorentz term accounts for the phonon excitation; thus, the total spectral weight has two contributions from the free carriers and the interband transitions including the phonon excitation, i.e.,

2 $$S{W}_{total}=S{W}_{Drude}+\,S{W}_{interband+phonon}.$$

**Figure 8:**
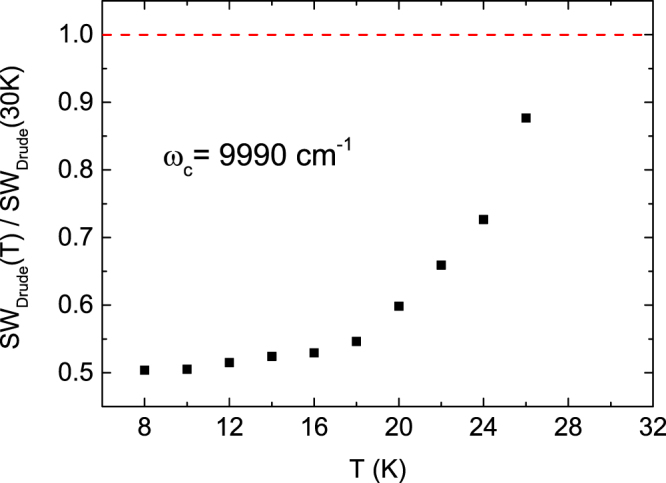
The temperature dependence of the spectral weight of the Drude terms normalized by its value at *T* = 30 K (normal state). When calculating the spectral weight, a cutoff frequency of *ω*_*c*_ = 9990 cm^−1^ was used. The spectral weight of the Drude terms at 300 K was evaluated to be 1.10 × 10^6^ Ω^−1^ cm^−2^.

**Figure 9:**
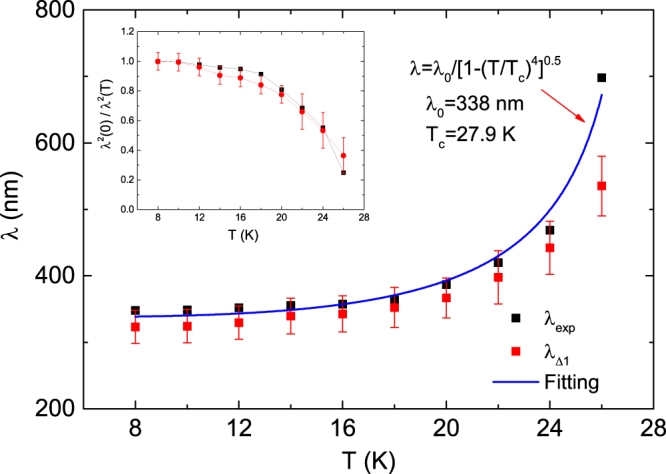
The temperature dependence of the penetration depth ${\lambda }_{exp}$ evaluated from eq. (3) using the data shown in Fig. 3(b) and the penetration depth ${\lambda }_{{\rm{\Delta }}1}$ evaluated from the missing area between the optical conductivity (dash-dotted blue lines in Fig. 4) calculated using the gap energy Δ_1_ and the optical conductivity calculated from the Drude response (solid green line in Fig. 2) using the larger plasma frequency ${\omega }_{P2}$ at 30 K in the (Ca_0.935_La_0.065_)_10_(Pt_3_As_8_)(Fe_2_As_2_)_5_ sample. The inset shows the total superfluid density and the superfluid density due to the smaller gap. The blue solid line is a line fit to a phenomenological equation, ${\rm{\lambda }}={\lambda }_{0}/[1-{(T/{T}_{c})}^{4}]$.

Therefore, the spectral weight due to only the free carriers is obtained by subtracting the Lorentz contribution including the phonon part from the total spectral weight. Figure 8 shows the temperature dependence of the spectral weight of the Drude terms normalized by its value at *T* = 30 K (normal state). A cutoff frequency of ${\omega }_{c}$ = 2000 cm^−1^ was used for calculating the spectral weight, which is high enough to cover all the spectral weight of the two Drude terms shown in Fig. 3. In the superconducting state, the spectral weight is suppressed by ~50%, indicating that approximately 50% of the conduction band electrons participate in the superconducting condensate. The London plasma frequency (${\omega }_{L})$ is derived as follows:

3 $${\omega }_{L}^{2}({\rm{\omega }}\to \infty )=\frac{{Z}_{0}}{{\pi }^{2}}\,{\int }_{0}^{\omega }[{\sigma }_{1}^{n}(\omega ^{\prime} )-\,{\sigma }_{1}^{s}(\omega ^{\prime} )]\,d\omega ^{\prime} $$

where ${\sigma }_{1}^{n}(\omega )$ is the optical conductivity spectrum for the normal state at *T* = 30 K, ${\sigma }_{1}^{s}(\omega )$ corresponds to the optical conductivity for the superconducting state and ${Z}_{0}$ is the vacuum impedance, which is equal to 377 Ω. By using the London plasma frequency, the penetration depth can be calculated from ${\rm{\lambda }}=1/2{\rm{\pi }}{\omega }_{L}$. Figure 9 shows the temperature dependence of the experimental penetration depth ${\lambda }_{exp}$ evaluated from eq. (3) using the data shown in Fig. 3(b) and the penetration depth ${\lambda }_{{\rm{\Delta }}1}$ due to the smaller superconducting gap contribution evaluated from the missing area between the optical conductivity (dash-dotted blue lines in Fig. 4) calculated using the gap energy Δ_1_ and the optical conductivity calculated from the Drude response (solid green line in Fig. 2) using a larger plasma frequency ${\omega }_{P2}$ at 30 K. The penetration depth ${\lambda }_{{\rm{\Delta }}1}$ due to the smaller gap Δ_1_ exhibits a smaller penetration depth and dominates the value of the penetration depth ${\lambda }_{exp}$ within the error bars. Moreover, the variation of the penetration depth with temperature is found to fit very closely to the relation ${\rm{\lambda }}={\lambda }_{0}/[1-{(T/{T}_{c})}^{4}]$ with ${\lambda }_{0}=338\,{\rm{nm}}$ and ${T}_{c}=27.9\,{\rm{K}}.$ The penetration depth ${\lambda }_{0}$ at 0 K and ${T}_{c}$ are consistent with the result obtained for optimally La-doped Ca10-3-8 samples^12^ and the result obtained from the electrical resistivity mentioned above, respectively. The inset of Fig. 9 shows the total superfluid density and the superfluid density due to the smaller gap. The total superfluid density is almost filled by the superfluid density due to the smaller gap despite its dirty limit; this occurs because the gap is formed by a larger Fermi surface. Furthermore, the two superfluid densities are flat in the low temperature regime, indicating an *s*-wave full-gap superconducting state^33^.

## Conclusions

We measured the optical reflectivity *R*(*ω*) for an underdoped (Ca_0.935_La_0.065_)_10_(Pt_3_As_8_)(Fe_2_As_2_)_5_ single crystal in the frequency range from 20 to 12000 cm^−1^ at 30 K (normal state) and obtained the sample optical conductivity ${\sigma }_{1}(\omega )$ using the K-K transformation. The optical conductivity ${\sigma }_{1}(\omega )$ is well fitted by the Drude-Lorentz model with two Drude components (${\omega }_{p1}=1446\,{{\rm{cm}}}^{-1}\,{\rm{and}}\,{\omega }_{p2}=6322\,{{\rm{cm}}}^{-1}$) and seven Lorentz components. The two Drude components strongly indicate the presence of multiple bands. The Lorentz fitting result indicates that bound electron bands near *E*_F_ are mainly dominated by a part of the Fe 3*d* bands and the Pt 5*d* bands. In the superconducting state, we also measured the *R*(*ω*) of the underdoped (Ca_0.935_La_0.065_)_10_(Pt_3_As_8_)(Fe_2_As_2_)_5_ single crystal for frequencies ranging from 20 to 600 cm^−1^ at various temperatures and obtained ${\sigma }_{1}(\omega )$. The ${\sigma }_{1}(\omega )$ data show the opening of a superconducting gap with a weaker second gap structure. The ${\sigma }_{1}(\omega )$ is well fitted by two superconducting gaps, with the magnitudes of the gaps estimated to be ${{\rm{\Delta }}}_{1}=\,30\,$ and ${{\rm{\Delta }}}_{2}=\,50$ cm^−1^, respectively, at *T* = 8 K, which both decrease with increasing temperature. The temperature dependence of the gaps was not consistent with one-band BCS theory but was well described by a two-band BCS model with interband interactions. The superfluid density calculated from the penetration depth indicated that the underdoped (Ca_0.935_La_0.065_)_10_(Pt_3_As_8_)(Fe_2_As_2_)_5_ compound shows a superconducting state with an s-wave full gap.

## Methods

Single crystals of (Ca_0.935_La_0.065_)_10_(Pt_3_As_8_)(Fe_2_As_2_)_5_ were grown by a Bridgman method with sealed molybdenum and boron nitride (BN). In the first step, precursors of CaAs, LaAs and FeAs were synthesized in evacuated quartz ampoules at 550, 800 and 1050 °C, respectively. In the second step, a mixture of the precursors and platinum was put into a BN crucible; then, this crucible was placed into a Mo crucible, with a Mo lid welded onto the crucible using an arc-welder in a high-purity Ar-gas atmosphere. In the final step, the entire assembly was slowly heated up to 1380 °C in a vacuum furnace, consisting of a tungsten meshed heater with a temperature stability of 0.1 °C, and was maintained at this temperature for 48 hours; afterwards, the assembly was moved slowly in a downward direction at a rate of 1.8 mm/hour for approximately 80 hours and, finally, slowly cooled down to room temperature.

To study the electronic structure in the normal state of (Ca_0.935_La_0.065_)_10_(Pt_3_As_8_)(Fe_2_As_2_)_5_, the optical reflectivity spectra *R*(*ω*) for the single crystal were measured in the frequency regions 70–12000 cm^−1^ and 20–150 cm^−1^, respectively, using a Michelson-type and a Martin-Puplett-type rapid-scan Fourier spectrometer at 30 K. To accurately obtain the absolute *R*(*ω*) values, an *in situ* Au overfilling method was adopted^34,35^.

In an attempt to estimate the fine temperature dependence of the superconducting gaps for (Ca_0.935_La_0.065_)_10_(Pt_3_As_8_)(Fe_2_As_2_)_5_, we carried out relative reflectivity measurements (RRM) in the frequency range from 20 to 600 cm^−1^ at varying temperatures below *T*_c_^36^, in addition to the absolute reflectivity measurements (ARM) mentioned above. By using this technique, we can precisely determine the reflectivity of a material over a relatively narrow temperature range without any physical displacement of the sample. Moreover, the data at each temperature are divided by the measured intensity at one specific arbitrary temperature *T*_0_, and then, each temperature is multiplied by the absolute reflectivity measured at *T*_0_. The reflectivity data obtained using this new method for the superconducting state below *T*_c_ coincided with the data at 30 K (normal state) near 600 cm^−1^; thus, the reflectivity data above 600 cm^−1^ in the superconducting state were replaced by the data at 30 K.
